# Editorial: Endocrine disruptors and diseases of brain and mind: past and prelude

**DOI:** 10.3389/fendo.2024.1362519

**Published:** 2024-01-17

**Authors:** Ashu Johri, Luca Roncati, Fernando Lizcano

**Affiliations:** ^1^ Independent Researcher, New York, NY, United States; ^2^ Department of Surgery, Medicine, Dentistry and Morphological Sciences with interest in Transplantation, Oncology and Regenerative Medicine, University of Modena and Reggio Emilia, Modena, Italy; ^3^ Centro de Investigacion Biomedica (CIBUS), Doctoral program in Biosciences, Universidad de La Sabana, Chía, Cundinamarca, Colombia

**Keywords:** endocrine disrupting chemicals (EDCs), organophosphate flame retardants (OPFRs), phthalates, neurodevelopment, autism, dementia, chorea, Fahr’s syndrome

## Perspective

Endocrine disruptors or endocrine disrupting chemicals (EDCs) are structurally diverse synthetic and naturally occurring compounds that interfere with the endocrine system with potentially adverse health outcomes. Relatively low concentrations of EDCs persist in the environment and produce long-term alterations percolating from generation to generations. Exposure to EDCs is associated with cancer, stillbirth or birth defects, developmental disorders, metabolic changes, and is increasingly suspected in the sporadic occurrences of neurological and neuropsychiatric disorders (1–5). Following is a brief summary of the articles that were published in this Research Topic.

## Organophosphate flame retardants in young population

Organophosphate flame retardants (OPFRs) are thermally stable organic compounds widely used in many housing materials and consumer products and were thought to be safer alternatives to other previously used versions of flame retardants. However, recent studies have linked these compounds to endocrine disruption, cardiovascular and neurodevelopmental abnormalities, and cancer. As part of a cohort study examining the adverse health effects of OPFRs in a broad range of young population (0-18 years old) in the Southern region of Taiwan, Chen et al. documented the urinary levels of both the parent compounds as well as primary metabolites of OPFRs. They reported that the presence of OPFRs and its metabolites was comparatively higher in newborns and toddlers, which they attributed to the longer time spent indoors for this age group, increasing the likelihood of exposure to house dust, foam, wooden and textile materials. Moreover, in the newborn group they found a negative correlation between some of the OPFR metabolites and birth outcomes such as birth length, birth weight, head circumference, chest circumference and abdominal circumference. However, they did not find a significant correlation between the urinary OPFRs and creatinine levels in the newborns. Although further studies are needed to determine the relationship between OPFRs and the susceptibility to negative health outcomes, this study further supports the usefulness of urinary levels of OPFRs and OPFR metabolites as biomarkers of exposure to OPFRs.

## Phthalates and neurodevelopment

Phthalates (phthalic acid diesters) are classified as EDCs, are ubiquitous pollutants, and have the ability to cross the placenta. This raises valid concerns for developmental effects from *in utero* exposure, considering the higher susceptibility of the fetus and child. Lucaccioni et al. performed careful and meticulous investigations regarding peri- and postnatal exposure to phthalates in an Italian pediatric cohort of full-term babies and their mothers. They examined the relationship between the urinary levels of phthalate metabolites and global development indices using the Griffiths Scales of Children Development (GSCD) at six months of age. Their studies showed that phthalate exposure is widespread in the area in which the study was undertaken. In line with previous findings, Lucaccioni et al. found negative associations of GSCD III scores such as language and hand-eye coordination in children with higher levels of certain phthalate metabolites. Intriguingly, they found a clear sex-related difference in the impact of phthalates on neurodevelopment. These findings assume significance in light of the previously published studies showing phthalate exposure is associated with a variety of neurodevelopmental outcomes, including autism, attention deficit hyperactivity disorder, reduced IQ, and reduced mental and psychomotor development, while some studies did not find any significant associations. Additional large-scale studies are needed to confirm these findings since humans are chronically exposed to phthalates during all stages of life.

## EDCs and autism

Cunha et al. examined the association between maternal exposure to EDCs during pregnancy and the risk of autism spectrum disorders (ASD) in children. Upon thorough research using defined criteria and screening for eligibility, data extraction, and assessment of bias, 27 observational studies were included assessing prenatal exposure to phthalates, polychlorinated biphenyls, organophosphate pesticides, phenols, perfluoroalkyl substances, organochlorine pesticides, brominated flame retardants, dioxins, and parabens. The number of examined children ranged from 77 to 1,556, the age at the assessment of autistic traits ranged from 3 to 14 years, and most studies assessed autistic traits using the Social Responsiveness Scale. Cunha et al. did not find an association between maternal exposure to specific EDCs and a likelihood of autistic traits in the progeny later in life. The authors, however, caution against interpreting this study as definitive evidence of no association between prenatal exposure to EDCs and autistic traits, citing the limitations of studies under examination such as representative exposure assessment, small sample sizes, inadequacy to assess sexually dimorphic effects, or the effects of EDC mixtures. Finally, this review could be helpful to guide future investigators in circumventing the limitations of the examined studies as outlined in this systematic review.

## Hormones and dementia

Life-long exposure to EDCs, especially estrogenic EDCs, contributes to the superfecta of metabolic syndrome that includes insulin resistance, type 2 diabetes mellitus, diabetes-related dementia and dyslipidemia, and Alzheimer’s disease (AD), which is sometimes dubbed as type 3 diabetes. Environmental estrogens and anti-androgens can interfere with normal pancreatic function, insulin signaling pathways, brain insulin resistance and dyslipidemia, leading to diabetes and related dementia. Takechi et al. highlight another dimension to this complex problem as they propose that dyslipidemia and increased plasma abundance of triglyceride rich lipoproteins (TRLs) in type 2 diabetes coalesce and bind amyloid-β peptide (TRL-Aβ) which in turn compromises cerebral capillary integrity. Further, heightened extravasation of TRL-Aβ precipitates neurovascular inflammation, resulting in neuronal demise and premature cognitive decline. These studies not only provide new insights for onset and progression of AD but could also potentially open-up new therapeutic avenues.

## Fahr’s syndrome

Fahr’s syndrome also known as primary familial brain calcification (PFBC), and familial idiopathic basal ganglia calcification (FIBGC) is a rare, dominantly inherited neurological disorder with abnormal deposits of calcium in the basal ganglia and the cerebral cortex of the brain. Nawaz et al. present the case report of a man who they diagnosed to be suffering from Fahr’s syndrome based on neuropsychological and neuroimaging findings. Based on their findings, they propose that autoimmune polyendocrine syndrome should be considered as the etiology of Fahr’s syndrome. This is also a first report of an unusual association of neuromyelitis optica spectrum disorder with Fahr’s syndrome.

## Chorea in endocrine diseases

Next in this topic, Zheng and Wu present a unique and comprehensive perspective on chorea as an unusual clinical manifestation of endocrine diseases such as thyroid, parathyroid, diabetes as well as electrolyte disturbances. Chorea is a rare disorder with abrupt, involuntary, dance-like movements and is usually associated with neurological disorders which is why endocrine disease-related chorea is often misdiagnosed. This review provides a detailed account of current knowledge on endocrine disease-related chorea, possible mechanisms involved and recommendations to aid in early diagnosis and treatment of this ailment.

## Future directions

In conclusion, our Research Topic presents a cautionary tale of past exposures to EDCs and long-term adverse effects of some of these compounds. Sometimes, it is worth revisiting the past to learn the lessons for future generations and in providing a safe and healthy environment for everyone. While technological advances have certainly made our lives easier, it has also taken a toll on overall general well-being of people. Relatively low concentrations of the EDCs are sufficient to produce long-term health problems, sometimes across generations. Thus precautions must be taken to avoid exposure to the toxic chemicals, whether it is through restricted use and safe disposal or by replacing them with safer natural alternatives. With the rising world population, there is obviously a crunch for natural resources and research must continue in this direction to attain sustainability. An important avenue could be the use of artificial intelligence (AI) in deciphering the potential interactions of chemicals with hormonal pathways and the use of adverse outcomes pathways (AOP) for the description of the effect of substances on biological pathways. This could save precious time and resources and help identify and root-out the potentially hazardous compounds from becoming a part of our everyday life.

## Author contributions

AJ: Conceptualization, Investigation, Project administration, Writing – original draft, Writing – review & editing. LR: Writing – review & editing. FL: Writing – review & editing.
